# Editorial: Challenges in Vaccinology

**DOI:** 10.3389/fimmu.2020.632537

**Published:** 2020-12-17

**Authors:** Ed C. Lavelle, Rita Carsetti, Winfried F. Pickl, Ursula Wiedermann

**Affiliations:** ^1^ Adjuvant Research Group, School of Biochemistry and Immunology, Trinity Biomedical Sciences Institute, Trinity College Dublin, Dublin, Ireland; ^2^ Immunology Research Area B-Cell Development Unit, Immunological Diagnosis Unit, Bambino Gesù Children Hospital, Rome, Italy; ^3^ Institute of Immunology, Center of Pathophysiology, Infectiology & Immunology, Medical University Vienna, Vienna, Austria; ^4^ Institute of Specific Prophylaxis and Tropical Medicine, Center of Pathophysiology, Infectiology & Immunology, Medical University Vienna, Vienna, Austria

**Keywords:** vaccine, systems biology, adjuvant, emerging infectious diseases, nosocomial infections, demographic changes, microbiome

The COVID19 pandemic has focused minds as rarely before on the vital contribution of vaccines to modern life. In addition to issues related to antigen identification, vaccine adjuvants, vectors and formulations, knowledge is increasing on how a range of factors including age, sex, comorbidities, and the microbiome can impact on responses to vaccines. In this issue, the wide breadth of outstanding issues and opportunities are raised relating to urgent vaccine needs, limitations of current approaches, and the potential of cutting edge technologies to facilitate new advances are highlighted.

These issues are clearly outlined in the review from Kennedy et al. where the great potential of ‘omics’ approaches supported by bioinformatics to provide insights into pathogen biology, host genetic diversity, and other factors to guide future vaccine research and development through the emerging field of ‘vaccinomics’ are outlined.

Even before the current SARS-CoV-2 pandemic, there have been a number of endemic and emerging viral pathogens for which vaccines still are urgently required. Esposito and Principi address Norovirus, among the most common causes of outbreaks of acute gastroenteritis and sporadic acute diarrhea episodes and the current status of vaccine development against the virus. The Arboviruses, Chikungunya, and Zika virus transmitted by Aedes mosquitoes are of increasing concern due to more widespread prevalence globally. There is promise regarding the feasibility of vaccination, but there are many challenges including in the design and location of phase III trials that are critically addressed by Schrauf et al. The seasonal variation in dominance of influenza virus strains and evolving subclades poses an enormous challenge for vaccinology. Influenza vaccine effectiveness is addressed by Redlberger-Fritz et al. who stress the vital importance of detailed genetic virus surveillance to address subtype specific vaccine effectiveness on an ongoing basis.

In addition to emerging pathogen threats for which no vaccines currently exist, antimicrobial resistance is a global emergency, and vaccination will have to play a part in dealing with its consequences. Rosini et al. address these challenges an the potential mitigation strategies, highlighting the remarkable projection that “In terms of magnitude, the economic impact of AMR is estimated to be comparable to that of climate global change in 2030.” The enormous challenge posed by nosocomial hospital-acquired bacterial infections presents a need for novel approaches including in the assessment of vaccine efficacy and the design of clinical trials (Bekeredjian-Ding). Careful stratification of patient groups may be critical to demonstrate efficacy where a universal vaccination approach is not feasible. Beyond infectious diseases, vaccines also have great potential to address chronic diseases. Allergy is considered an epidemic, which affects almost 30% of the population. Innovative allergen immunotherapy approaches offer the promise of reducing allergic symptoms, and potentially vaccination could in future be used in a prophylactic manner to prevent allergies (Tulaeva et al.).

The importance of targeting specific patient groups with tailored vaccine strategies is becoming increasingly clear. Given demographic changes, the percentage of older individuals across the globe will increase over the coming decades. Given the toll taken by infectious diseases in terms of morbidity and mortality and the lower efficacy of a number of vaccines including those against influenza in this older patient cohort, innovative approaches are required to develop more effective vaccines for this group (Wagner and Weinberger). There is significant promise in this regard with evidence with some vaccines particularly against herpes zoster that innovative adjuvant approaches may help to increase vaccine efficacy.

Immune responses to many vaccines have been found to be impacted by sex, and the field of sex differences in vaccine efficacy is likely to expand over the coming years providing valuable insights for future vaccine design and implementation. In a study on immune responses to a tick borne encephalitis booster vaccination (Garner-Spitzer et al.), cellular and humoral responses, and systemic side effects to the vaccine were affected by obesity and biological sex. Obesity is increasingly recognized as a key factor to address in terms of its impact on the magnitude and type of vaccine induced innate and adaptive immune responses. The impact of another comorbidity, inflammatory bowel disease, is considered by Lenti et al. in the context of susceptibility to encapsulated bacteria, and the importance of addressing the effectiveness of existing vaccines for encapsulated bacterial pathogens in these patients is highlighted.

There have been great advances in antigen discovery, characterization, and optimization over recent years spurred by developments in omics technologies, bioinformatics, and the emerging field of structural vaccinology. The concept of ‘computational vaccinology’ is outlined by De Groot et al. who explain the underlying concepts of how these approaches allow rational antigen discovery and design and a toolkit for vaccine design named iVAX. Tobias et al. present a novel approach for B cell based cancer vaccines where vaccination with mimotopes of immune checkpoint inhibitors alone or together with tumor-specific vaccines promoted enhanced anti-tumor immunity. Bettencourt addresses the need for an effective malaria vaccine and, based on the documented protective efficacy of irradiated sporozoites, outlines the potential of technologies, particularly immunopeptidomics, to identify liver stage antigens for inclusion in future vaccines.

No vaccines are available for Schistosomiasis, which is a significant public health problem over much of the globe. Hernández-Goenaga and colleagues propose Kunitz-type serine protease inhibitors as possible vaccine targets. Using RNA-seq, bioinformatics to predict T- and B-cell epitopes, chemical synthesis, and adjuvant formulation the authors demonstrated a degree of protective efficacy of the selected antigens against experimental Schistosomiasis in mice (Hernández-Goenaga et al.).

The majority of influenza viruses are derived from virus growth in embryonated eggs, but this process is time consuming with potential limitations in terms of antigenicity and challenged by highly pathogenic avian influenza viruses. Cell culture based approaches have been used to produce antigen and are already included in a licensed vaccine. Here, Jawinski et al. propose the use of the ciliated protozoan *Tetrahymena thermophila* to express influenza haemagglutinin and demonstrate the immunogenicity of the expressed protein particularly when combined with a nanoparticle adjuvant. The potential of an adjuvanted Nucleotide exchange factor, GrpE, a highly conserved heat shock protein to induce protective immunity against genital tract infection with the bacterial pathogen *Ureaplasma urealyticum* in mice was demonstrated by Tang et al.

While antigen discovery and optimization is critical to ensure the specificity of adaptive immune responses, the magnitude and type of such responses is principally directed by adjuvant induced innate immunity with subunit vaccines or microbial factors in the case of whole cell, inactivated/split virus, or attenuated vaccines. Adjuvants can also facilitate enhanced responses in specific target groups such as the elderly or neonates. In this context, germinal center activation is limited in neonates which can lead to reduced generation of antibody secreting cells and transient antibody responses. Arandottir Pind et al. compared a number of clinically applied and experimental adjuvants (LT-K63, mmCT, MF59, IC31, and alum) to enhance responses of neonatal mice to a pneumococcal conjugate vaccine Pnc1-TT. LT-K63, mmCT, MF59, and IC31 were more effective than alum in promoting these responses, offering promise for improved neonatal vaccine strategies in future. This team carried out a detailed analysis on how the adjuvant LT-K63 enhanced antibody responses, demonstrating upregulation of tumor necrosis factor receptor superfamily members involved in the initiation and maintenance of antibody responses (Aradottir Pind et al.).

Stefan Kaufmann addresses the current state of the art with prophylactic and therapeutic tuberculosis vaccine approaches including the range of vaccine strategies under evaluation, including adjuvanted subunits, viral vectored vaccines, and BCG based constructs in clinical trials. The major endpoints for clinical trials: prevention of infection, prevention of disease, and prevention of recurrence are also highlighted (Kaufmann). Prime-boost regimes are being widely tested in the context of TB vaccination and also in many other settings. Sanchez Alberti et al. present data following DNA vaccine prime and adjuvanted subunit antigen booster vaccination against *Trypanosoma cruzi*, showing that formulations which primed polyfunctional CD4 and CD8 responses most effectively protected against challenge in a murine model

While the majority of vaccines are delivered by intramuscular vaccination, there is a desire to develop more patient friendly vaccine administration routes. Transdermal vaccination has many advantages including the targeting of an environment rich in antigen presenting cells. Pielenhofer et al. address the potential of particle based vaccine systems to overcome challenges related to the transcutaneous route and promote stronger immune responses.

Addressing the mechanisms by which vaccines activate innate immune responses and promote protective adaptive immunity is critical to helping us understand how vaccines work and in providing targets to improve future vaccine efficacy. Abreu et al. assessed serum IgA responses following vaccination with split influenza vaccines across number of influenza seasons. The authors concluded that influenza specific IgA antibodies are an important immune correlate that should be considered in addition to IgG. Assessment of IgA responses to the 23 valent pneumococcal polysaccharide vaccine is proposed by Pulverenti et al. as a prognostic marker in common variable immunodeficiency (CVID). The authors found that inability to mount an IgA-mediated response against the polysaccharide antigens or maintain the antibody response over time identified poor IgA CVID responders with severe immunological impairment, greater risk of co-morbidities, and poor prognosis.

The RTS,S/AS01 vaccine induces partial protection against *Plasmodium falciparum* but determinants of vaccine induced protection are needed. Based on data from host blood transcriptomes, Du et al. propose the transcript ratio, MX2/GPR183 to discriminate protected from non-protected individuals. The results indicate a role for interferon and oxysterol signaling in the vaccine mode of action. There is a need for safe and effective approaches to promote stronger cytotoxic T cell responses for both infectious disease and cancer vaccines. The bacterium, *Listeria monocytogenes* is an effective vector for vaccine antigens, triggering strong CD8 responses following administration (Rana et al.). The authors demonstrated roles for caspase 1/11 and RIPK3 in dictating the quality of CD8 responses induced by the vaccine.

In summary, these articles encapsulate the current dynamism of the vaccine field; highlighting major current challenges and those on the horizon in addition to exciting developments in antigen and adjuvant discovery. Increased knowledge of vaccine mode of action and how this is impacted by factors including age, sex and health status will be critical in making further advances and moving in some cases toward more stratified vaccination regimes.

## Author Contributions

All authors listed have made a substantial, direct, and intellectual contribution to the work and approved it for publication.

## Conflict of Interest

The authors declare that the research was conducted in the absence of any commercial or financial relationships that could be construed as a potential conflict of interest.

